# Worker Stress, Burnout, and Wellbeing Before and During the COVID-19 Restrictions in the United Kingdom

**DOI:** 10.3389/fpsyg.2022.823080

**Published:** 2022-04-05

**Authors:** Diane Pelly, Michael Daly, Liam Delaney, Orla Doyle

**Affiliations:** ^1^Department of Economics, University College Dublin, Dublin, Ireland; ^2^Department of Psychology, Maynooth University, Maynooth, Ireland; ^3^Department of Psychological and Behavioural Science, London School of Economics and Political Science, London, United Kingdom

**Keywords:** COVID-19 restrictions, lockdown, homeworking, subjective wellbeing, stress, burnout, mental health

## Abstract

COVID-19 created a transformational shift in the working environment for much of the labour force, yet its impact on workers is unclear. This study uses longitudinal data to examine the wellbeing of 621 full-time workers assessed before (November 2019–February 2020) and during (May–June 2020) the first lockdown in the United Kingdom. We employ fixed effects analyses to investigate the impact of the restrictions and mandatory homeworking on cognitive, emotional, and psychological wellbeing. Within the sample, the rate of full-time homeworking increased from 2 to 74% between waves. We identify significant changes in 9 of the 15 measures assessed, with a general pattern of improvements in wellbeing during lockdown. Overall levels of stress, self-rated mental health, positive emotions and life and job satisfaction are not adversely affected by the restrictions. There is a reduction in the burnout symptoms of disengagement (−0.13 sd) and exhaustion (−0.20 sd) and in the frequency with which negative emotions are experienced at work (−0.15 sd). Workers feel more autonomous (+0.09 sd), closer to their co-workers (+0.10 sd), and more attached to their organisations (+0.19 sd). However, homelife satisfaction declines (−0.11 sd). These findings highlight the possibility that the COVID-19 pandemic and large-scale transition to homeworking was associated with unchanged or improved worker wellbeing. This study has important implications for governments and employers regarding a global shift to homeworking.

## Introduction

The COVID-19 restrictions have resulted in a major restructuring of work and home lives, with potential consequences for mental health and wellbeing. A burgeoning interdisciplinary literature has begun to examine the impact of this unprecedented shock, yet many studies are limited to data collected after the onset of the pandemic and/or utilise a narrow set of outcome measures. The current study contributes to this literature by producing a rich account of the lived experiences of United Kingdom workers, surveyed before and during the imposition of the COVID-19 restrictions. Specifically, a pre-post pandemic design is used to estimate the effects of “lockdown” and mandatory homeworking on general and work-related stress, burnout and wellbeing across a wide range of measures.

**FIGURE 1:**
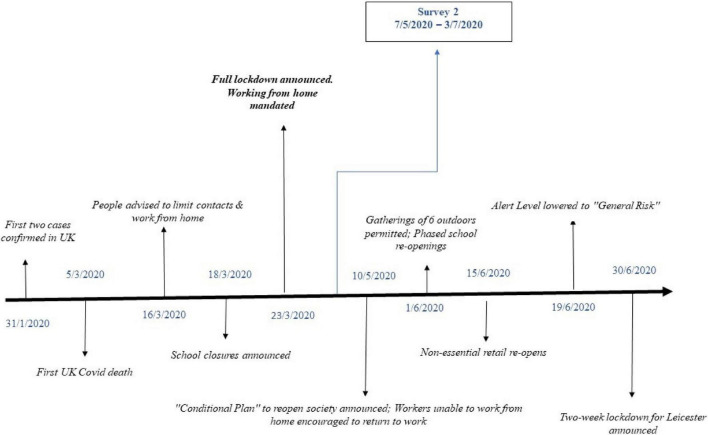
COVID-19 restrictions in the United Kingdom: Timeline (January 2020–July 2020).

On the 23rd of March 2020, the United Kingdom Prime Minister announced a statutory ban on leaving the home, including commuting to work, unless “absolutely necessary.” The United Kingdom remained in lockdown for 11 weeks, with a phased re-opening commencing in June 2020. Figure 1 depicts this timeline. The mental health and wellbeing effects of pandemics, including COVID-19, have been examined across an extensive set of studies (e.g., Lau et al., 2008; Brooks et al., 2020), however, these studies rely predominantly on cross-sectional designs, without pre-shock baseline assessments. Prior studies have also tended to use narrow, single-item measures of subjective wellbeing. While some longitudinal studies initiated during COVID-19 have investigated changes in mental health among the general population (e.g., Daly et al., 2020; Pierce et al., 2020) or on groups of interest such as frontline workers (e.g., Cabarkapa et al., 2020), the psychological impact of COVID-19 on general workers has not been investigated in depth. Where workers have been the primary focus (e.g., Bell and Blanchflower, 2020), the emphasis is often on the distributional effects of COVID-19 in terms of unemployment and income losses, rather than on subjective wellbeing. In this study, we examine full-time workers who were subjected to a dual shock–the impact of COVID-19 in communities across the United Kingdom and, for many workers, a radical change in where and how they work.

This study makes a unique contribution to the COVID-19 literature by investigating multiple facets of wellbeing including general and work-related cognitive, emotional and psychological dimensions. We also explore heterogeneity and investigate whether the restrictions differentially impact the wellbeing of homeworkers (77%), women (64%), and parents of young children (24%). The study also contributes to the homeworking literature by using the Day Reconstruction Method (“DRM”) (Kahneman et al., 2004) to capture, for the first time, the lived reality of homeworking before and during the pandemic. In doing so, it sheds light on the homeworking experiences of workers who may not have chosen to work from home and who may be combining work, alongside increased caring and/or home-schooling responsibilities.

### COVID-19 and Life Satisfaction, Happiness and Stress

Since the onset of COVID-19, a number of studies have examined its potential psychological effects on distress and mental health symptoms. Depression, anxiety and stress are common global reactions to the early stages of the pandemic (Rajkumar, 2020; Wang et al., 2020), with women and young adults faring particularly adversely (Pierce et al., 2020). There is, however, emerging evidence of psychological adaptation in the aftermath of the first wave in the United Kingdom (Fancourt et al., 2020; Daly and Robinson, 2021a), with mental health symptoms spiking sharply at the start of lockdown, before recovering in June and July 2020. Daly and Robinson (2021b) reveal similar findings using nationally representative United States data.

A limited number of pre-post pandemic studies examine the trajectory of wellbeing outcomes other than psychological distress. Entering lockdown is associated with reduced life satisfaction in Italy (e.g., Ruggieri et al., 2021). In the United Kingdom, Fancourt et al. (2021) show that, while average life satisfaction dips prior to lockdown, it increases after lockdown is announced and stabilises by the end of May 2020, albeit at a lower level. This lends support to an adaptation theory (Diener et al., 2009). Globig et al. (2020) show that the happiness of United States respondents surveyed at the start of the pandemic returns to baseline levels within 1 month. Fancourt et al. (2020) suggest that lockdown is not necessarily a negative experience for everyone, with 33% of respondents (mainly higher earners or people living with others) “enjoying” it. Similarly, De Vries et al. (2021) use longitudinal data to show that 15% of Dutch respondents feel more optimistic and find life more meaningful during lockdown, a finding they attribute to the “simplifying” effect of the pandemic. Recchi et al. (2020) report higher wellbeing for French respondents from higher socio-economic backgrounds, a finding they attribute to favourable social comparisons. Lockdown is, however, also associated with increased domestic discord (e.g., Luetke et al., 2020) and stress (Beland et al., 2020) and reduced wellbeing of parents and women (Huebener et al., 2021).

Very few studies focus exclusively on the impact of the COVID-19 restrictions on *worker* wellbeing. One notable exception is Zacher and Rudolph’s (2021) study which reveals a decrease in life satisfaction and happiness (global positive affect) amongst German workers during the early stages of the pandemic. Against expectations, they also find a reduction in negative affect, a finding they attribute to coping strategies, particularly the use of social supports. They speculate that this result may be driven by their reliance on measures of high-activation negative emotions (e.g., “upset”) and that unobserved low-activation negative emotions (e.g., “despondent”) may have increased during lockdown. The present study advances this work by analysing both high and low activation emotions, allowing us to provide a more nuanced insight into the affective mechanisms at work.

### COVID-19 and Homeworking

For a large portion of United Kingdom workers, lockdown triggered a sudden switch to homeworking for the first time. While homeworking is usually positively associated with higher job satisfaction and organisational commitment under “normal” circumstances, the links between homeworking, stress, emotional wellbeing, and burnout remain contested (e.g., Charalampous et al., 2019; Oakman et al., 2020).

In the context of COVID-19, the results are inconclusive. Ipsen et al. (2021), using cross-sectional data on the early lockdown experiences of 5,748 knowledge workers from 29 European countries, shows that, on balance, homeworking during lockdown was experienced positively. Weitzer et al. (2021) find that homeworking during lockdown in Austria is associated with improved quality of life for workers of all age groups and levels of education. In total, 39% of Moretti et al.’s (2020) respondents report feeling less stressed when working from home relative to their pre-lockdown place of work. While Kunze et al. (2020) find that most participants wish to continue homeworking, they also report a significant association between homeworking during the pandemic and excessive workloads, resulting in increased exhaustion. Sato et al. (2021) find a negative association between switching to homeworking and developing depressive symptoms for women, whereas Lyttelton et al. (2020) find that homeworking mothers feel anxious and depressed more often than homeworking fathers. Xiao et al. (2021) report an association between transitioning to homeworking and decreased physical and mental wellbeing in the United States. Finally, Möhring et al. (2021) find no changes in homelife or job satisfaction amongst German workers who switch to homeworking during COVID-19. Whilst these studies provide an important insight into the experience of homeworking during the pandemic, they rely exclusively on cross-sectional data collected after the onset of COVID-19. The present study addresses this limitation.

### Measuring COVID-19 Related Changes in Stress, Burnout, and Wellbeing

The multi-dimensionality of worker wellbeing is well documented (see Linley et al., 2009; De Simone, 2014). Thus, rather than relying on measures of general psychological distress or life satisfaction, we utilise a wide range of measures which capture changes in general wellbeing, as well as changes in work-related satisfaction, emotions, sense of purpose and meaning, stress, burnout and psychological wellbeing associated with entering lockdown. In doing so, we acknowledge the documented need for workers to feel close to their colleagues (relatedness) and to achieve “mastery” over their working environment through goals consistent with their sense of self (autonomy) and ability (competence) (Ryff, 1989; Reis et al., 2000), in order to fulfil their potential. Optimal performance also requires workers to feel engaged, a state which arises when they experience high-activation positive emotions and find their work absorbing (Csikszentmihalyi, 1990), worthwhile (Seligman, 2018), and positively challenging (Bakker and Demerouti, 2008). Workers who experience low pleasure and activation may experience occupational burnout, a state characterised by physical and emotional exhaustion and by disconnectedness. A recent meta-analysis by Shoman et al. (2021) highlights excessive job demands and negative work attitudes as key predictors of occupational burnout. There is also evidence that workers from particular sectors (e.g., teachers) are more likely to experience burnout and that training courses and policies aimed at boosting workers’ internal resources and abilities to cope with work-related stress and emotional demands (adaptive coping strategies) may render them less susceptible to burnout (Pishghadam et al., 2021; Shoman et al., 2021).^1^

While worker wellbeing studies predominantly rely on single-item job satisfaction scales, emotional wellbeing (affective) measures can be “global” or “experiential.” Global measures capture workers’ beliefs about the typical, overall patterns of emotions experienced at work on a remembered basis (Bakker and Oerlemans, 2011), whereas experiential measures capture momentary affective states triggered by changes in external circumstances as they occur (e.g., who the worker is with at the time). Despite evidence that these measures are differentially determined (Hudson et al., 2016), global measures dominate the COVID-19 studies discussed above. Given the potential for the restrictions to temporarily disrupt the work context, we assess experiential affective measures as well as global measures in order to fully capture COVID-19 related changes in emotional wellbeing.

In contrast to the majority of COVID-19 studies, we examine within-person changes in cognitive, emotional, and psychological measures of wellbeing. Our use of pre- and during pandemic data enables us to control for individual differences in workers’ pre-pandemic wellbeing levels and to capture variations in patterns of wellbeing changes associated with entering the first period of COVID-19 restrictions. Given emerging evidence of significant heterogeneity in how lockdown is experienced by different categories of workers, we also examine between-person differences, focusing on women, parents of young children and workers who worked from home during lockdown.

In line with existing COVID-19 research, we expect the imposition of pandemic related restrictions to be associated with a reduction in life and home-life satisfaction and with an average overall decrease in positive emotions and an increase in negative emotions, in particular anxiety and stress. Given the well-documented links between global measures and enduring life circumstances and the relatively short gap between the two surveys, we hypothesise that experiential affective measures will be more sensitive to COVID-19 induced changes than global measures of wellbeing. In line with existing research, we expect mental health to be adversely affected by COVID-19 restrictions, with women and parents likely to be worse affected given the imposition of additional caring and home-schooling burdens. Given that non-homeworkers are mostly essential front-line workers and that many homeworkers may have been forced to switch to a new (and not necessarily, preferred) way of working for which they were ill-prepared, we also expect to see decreases in job satisfaction and increases in work-related stress and psychological distress. However, while we expect the overall impact of COVID-19-related restrictions on our sample to be detrimental, we acknowledge the potential mitigating factor of sample composition, which we hypothesise may partially offset the anticipated overall average negative effect of the pandemic on the sample.

The remainder of the paper is organised as follows: Section “Materials and Methods” describes the data and outlines the empirical strategy and robustness checks. Section “Results” presents the results. Section “Discussion” discusses the results and concludes.

## Materials and Methods

### Data and Sample

We collected longitudinal panel data from 621 full-time workers. Participants were sourced through Prolific Academic, a specialist academic research survey-panel provider, and were compensated for their time.^2^ The wave one survey was completed online by 994 workers based in the United Kingdom between 25/11/2019 and the 19/2/2020.^3^ 1,514 Prolific panel members met the pre-screening criteria and were invited by Prolific to participate in the survey. Of these, 994 panel members elected to participate in the survey, corresponding to a response rate of 65.6%. The wave two survey was restricted to workers who had participated in wave one. Matched data was collected from 741 respondents between 7/5/2020 and 3/7/2020 2020 (response rate of 75%).

The time period between the two data collection points ranged from approximately two and a half to 7 months, with an average gap of approximately four and a half months. The distribution of responses by month is graphed in Supplementary Figure 1. Figure 1 provides some additional background context on the pandemic situation in the United Kingdom at the time of the study in the form of a ‘COVID timeline’. While the majority (84%) of wave one responses were collected between November 2020 and January 2021, a period in which the virus had not yet reached the United Kingdom, 97 (16%) wave one responses were collected in February 2020, after the first two cases had been confirmed in the United Kingdom on January 31 but prior to the announcement of the first COVID death in the United Kingdom on March 5. All of the wave two responses were collected after full lockdown and mandatory homeworking was announced on March 23. 94% of wave two responses were obtained in May, 5% in June and just one response in July. The majority of wave two responses were collected during full lockdown, on the 7th--8th of May, prior to the publishing of the Conditional Plan to re-open society on May 10. Twenty-five responses were obtained between the 1st and 19th of June, a period which coincides with lockdown easing, including phased school and non-essential retail re-openings. Just eight responses were obtained on the 19th of June after the risk alert level was lowered to ‘‘general risk.’’^4^

The sample intentionally targets full-time workers. Pre-screening criteria were used to recruit participants between 18 and 65 years old, who were engaged in full-time paid employment for more than 2 months, in organisations with 5 or more workers, for at least 21 hours per week. Shift-/part-time and self-employed workers were excluded to reflect our focus on full-time workers and due to evidence that these groups experience systematically different health patterns (Reutrakul and Knutson, 2015). We excluded 120 participants from the final estimation sample as they were no longer engaged in paid work in wave two.^5^ Thus, the final estimation sample comprises 621 full-time workers who were present and employed in wave one and two. Wheatley’s (2021) study of the United Kingdom homeworking population using the Understanding Society dataset suggests that our sample is representative of the pre-COVID-19 homeworking population, which is more likely to be middle-aged, highly qualified, living with children and on a permanent contract. However, our sample contains a higher proportion of females and university graduates. Supplementary Table 2 compares the key demographic variables of our sample to that used by Wheatley (2021).

The descriptive statistics are set out in Table 1. Prior to COVID-19, just 2% of the sample worked from home full-time, which is in line with Wheatley (2021). In total, 17% homeworked “frequently” (at least 4 days per month), 13% homeworked “sometimes” (less than 1 day per month but more than 4 days per year), and 18% homeworked “occasionally” (less than 4 days per year). In total, 50% of participants “never” worked from home. By wave two, a dramatic shift to homeworking had occurred, with 74% homeworking on a full-time basis and 3% on a part-time basis. A total of 23% continued to work from their pre-COVID-19 location. In line with recent research (e.g., Adams-Prassl et al., 2020), workers on high (>£3,000 per month) salaries (86% vs. 72%; *p* = 0.014) and university graduates (82% vs. 59%, *p* < 0.001) are more likely to work from home during wave two.

**TABLE 1:** Personal and work-related characteristics.

	% Mean (*N* = 610–621*)*
**Gender**	
Female	64.0%
Male	35.5%
Other/Prefer not to say	0.5%
**Citizenship**	
British	93.7%
Northern Irish	1.9%
Other	4.4%
**Ethnicity**	
White	91.7%
Asian	3.5%
Black	2.4%
Other	2.4%
**Relationship status**	
Single/Divorced/Widowed	25.6%
In a relationship	74.4%
**Education**	
No formal education/Lower secondary	6.1%
Higher secondary	13.9%
Cert/Diploma	6.6%
Technical/Vocational	10.7%
Undergraduate	41.6%
Post-graduate	21.1%
**Age**	38.3
**Parental status**	
Parent	50.3%
Non-parent	49.7%
**Parent by age category**	
Under 5s	17.8%
5–12	20.4%
13–18	23.1%
Over 18	19.7%
**Living on their own**	
Yes	13.1%
No	86.9%
**Living with children**	
Yes	52.2%
No	47.8%
**Net monthly household income**	
<£1,000	1.5%
£1,000–£2,000	25.4%
£2,000–£3,000	30.8%
£3,000–£4,000	32.6%
>£4,000	9.7%
**Physical health** (1 = “Very bad”; 5 = “Very good”)	“Good” (53.4%)
**Effect of COVID-19 on income**	
No effect	56.7%
Financially worse off	31.3%
Financially better off	12.0%
**Physical health condition** (Wave 1 only)	
Yes	23.1%
No	76.9%
**Contracted COVID-19**	0.8%
**Quarantining or showing COVID-19 symptoms**	5.8%
**Mental health** (1 = “Very Bad”; 5 = “Very Good”)	“Good” (40.9%)
**Mental health condition** (Wave 1 only)	
Yes	23.6%
No	76.4%
**Contract type**	
Permanent	95.6%
Temporary/Fixed-term	4.4%
**Seniority** (0 = “Most junior”; 5 = “Most senior”)	3 (32.7%)
**Tenure**	
<5 years	50.6%
5–10 years	22.7%
>10 years	26.7%
**Pay-rise in the previous 12 months**	
Yes	53.9%
No	46.1%
**Net monthly salary**	
<£1,000	4.2%
£1,000–£2,000	52.6%
£2,000–£3,000	30.1%
£3,000–£4,000	8.7%
>£4,000	4.4%
**Hours worked previous month**	
Wave 1	158
Wave 2	142
**Sector**	
Private	60.3%
Public	39.7%
**Industry**	
Admin, IT, and Telecoms	12.0%
Arts/Entertainment/Tourism	2.6%
Construction	3.1%
Education and Childcare	14.3%
Food	2.4%
Healthcare	10.6%
Manufacturing	9.7%
Civil Service and Local Government	2.3%
Other Services	3.2%
Professional Services/Finance and Insurance	18.0%
Publishing/Media	1.8%
Retail	8.8%
Social Services and Law Enforcement	4.4%
Agriculture/Forestry/Fishing	0.5%
Transportation/Wholesale and Warehousing	3.3%
Utilities	2.3%
**Organisation size**	
Micro (<10 employees)	3.7%
Small (<50)	12.0%
Medium (<250)	19.0%
Large (>250)	63.8%
Dont Know	1.5%

### Measures

We employ 15 outcome variables to estimate the effect of the COVID-19 restrictions on worker wellbeing. Given that only two independent variables contain more than 31 missing observations, we adopt a complete case approach to missing values.^6^ A description of all variables is provided in Supplementary Table 3.

#### Cognitive Measures (3 Outcomes)

*Life satisfaction* is a global evaluative judgement made by an individual about the overall state of her/his life using a 0--10 scale. An identical format is used to measure workers’ *homelife satisfaction* and *job satisfaction*.^7^

#### Emotional Measures (5 Outcomes)

*Global emotional wellbeing* is measured using the **Institute of Work Psychology (IWP) Multiaffect Indicator** (Warr and Parker, 2010). Respondents indicate the extent to which they experienced 16 emotions (8 negative, 8 positive) at work during the past month (1 = “*Never*” and 7 = “*Always*”). For ease of comparison with the DRM, scores are recoded using a 0–6 scale Emotions are evenly split between high activation (e.g., “*excited*”) and low activation (e.g., “*depressed”*) emotions. Global positive (negative) affect is the mean of the 8 positive (negative) feeling scores. Cronbach’s alpha for wave one/wave two positive and negative affect are 0.894/0.903 and 0.926/0.923, respectively.

*Experiential emotional wellbeing* is measured using the **Day Reconstruction Method (DRM)** (Kahneman et al., 2004). Workers use diary entries to “reconstruct” 3 consecutive “episodes” from the previous working day. The time-of-day starting point for the episodes is randomly generated. Participants record when each episode started and ended; where they were; who they were with and what they were doing. They then rate the extent to which they experienced 16 emotions (the same used to measure global affect) during this episode, where 0 = “*Did not experience that feeling at all*” and 6 = “*That feeling was an important part of the experience*.” Average experiential positive and negative affect are the mean positive and negative scores for the 3 combined episodes, after 27 observations containing missing values are excluded. Cronbach’s alpha scores for wave one/wave two positive and negative experiential affect are 0.757/0.910 and 0.841/0.845, respectively.

*Affective commitment*, or the extent to which workers feel emotionally bound to their organisations, is measured using Meyer and Allen’s (1997) 6-item **Affective Commitment Scale.** Workers rate their agreement with 6 statements (3 positive, 3 negative), e.g., “*I do not feel like ‘part of the family’ at my organisation*,” where 1 = “*Strongly Disagree*”; 5 = “*Strongly Agree*.” Average commitment is the mean of the 6 scores, with reverse scoring applied to negative items. Cronbach’s alpha is 0.886/0.895 (wave one/wave two).

#### Psychological Measures (7 Outcomes)

*Burnout (disengagement* and *exhaustion*) is measured using Demerouti and Bakker’s (2008) validated (Halbesleben and Demerouti, 2005) 16-item **Oldenburg-Burnout Inventory (OLBI)**.^8^ Respondents use a 1–4 scale to rate their level of agreement with 8 negative and 8 positive statements, e.g., “*During my work, I often feel emotionally drained.*” Cronbach’s alpha is 0.898/0.894 (wave one/wave two). *Work-related stress* is measured using a 1–5 scale, where 1 = “*Not at all Stressful*”; 5 = “*Extremely Stressful.*” Workers also detail sources of work-related stress (e.g., “*job security*”). The extent to which workers’ needs for *relatedness* (feeling connected to co-workers*), competence* (feeling capable of attaining desired work-related goals) and *autonomy* (feeling that work is compatible with self-identity) are met is assessed using the 21-item Basic **Psychological Needs Satisfaction at Work Scale** (Deci et al., 2001). Respondents use a 1–7 scale to rank the trueness of statements, e.g., “I *really like the people I work with.*” Cronbach’s alpha is 0.873/0.874 (relatedness), 0.728/0.703 (competence), and 0.678/0.659 (autonomy). Finally, mental health is measured using a single item five-point scale (1 = “*Very Bad*”; 5 = “*Very good*”).

### Analyses

Using an approach similar to Pierce et al. (2020), we estimate changes in the wellbeing of worker *i* at time *t* (*Y*_it_) associated with entering lockdown using the equation:


(1) $$Y_{it}={\text{β}}_{0}+{\text{β}}_{1}wave_{i}+u_{i}+{\text{ε}}_{it}$$

where β*_0_* is the time-invariant intercept which is correlated with observed explanatory variables; *wave*_*i*_ is a dummy variable that takes the value 1 for wave two (May–June 2020) and 0 for wave one (November 2019–February 2020); *u*_*i*_ captures the individual fixed effects and ε*_*it*_* denotes independent and identically distributed time-varying random shocks. The parameter β*_1_* captures the baseline difference in *Y*_*i*_ between wave one (pre-lockdown) and wave two (during-lockdown). A fixed effects model is used given the high probability of unobserved characteristics confounding the relationship between COVID-19 restrictions and wellbeing (e.g., gender differences in the division of childcare). Sensitivity analyses, where the main analysis is re-estimated using OLS and a random effects models, reveal no material differences between the fixed effects and alternative approaches. These results are reported in Supplementary Table 4.

We first estimate within-person changes in wellbeing between wave one and two. Time-varying control variables are not included due to the short time gap between the two surveys which limits variation over time (e.g., education, number of children). In addition, many of the time-varying variables (e.g., income or physical health) are potential mechanisms or outcomes of the COVID-19 restrictions in their own right, therefore it is not appropriate to control for them in the wellbeing equations (cf. Pearl, 1999, 2014). We also investigate heterogeneity regarding the impact of entering the period of COVID-19 restrictions by interacting the wave variable with homeworking status (Eq. 2), gender (Eq. 3), and parental (young child) status (Eq. 4). Thus, we estimate the following three equations:


(2) $$Y_{it}={\text{β}}_{0}+{\text{β}}_{1}wave_{i}+{\text{β}}_{2}WFH_{i}+{\text{β}}_{3}wave_{i}\;*WFH_{i}+u_{i}+{\text{ε}}_{it}$$

(3) $$Y_{it}={\text{β}}_{0}+{\text{β}}_{1}wave_{i}+{\text{β}}_{2}Gender_{i}+{\text{β}}_{3}wave_{i}*Gender_{i}+u_{i}+{\text{ε}}_{it}$$

(4) $$Y_{it}={\text{β}}_{0}+{\text{β}}_{1}wave_{i}+{\text{β}}_{2}Parent_{i}+{\text{β}}_{3}wave_{i}*ParentU13_{i}+u_{i}+{\text{ ε}}_{it}$$

where *WFH*_*i*_ is a binary variable that captures homeworking status. ‘‘Non-homeworkers’’ (coded 0) comprise workers who report working outside the home during lockdown and ‘‘homeworkers’’ (coded 1) comprise workers who work from home to any extent during lockdown.^9^ β*_2_* captures the baseline difference in *Y*_*i*_ between workers who are homeworking or not during lockdown. β*_3_* captures the interaction between entering lockdown and homeworking. Equation 3 examines heterogeneity by gender. *Gender*_*i*_ is a binary variable, coded 0 for women and 1 for men. Six participants who identify as “non-binary/other” are omitted. β*_3_* captures the interaction between entering lockdown and gender. Finally, Eq. 4 examines the extent to which lockdown differentially impacts parents of young children. *ParentU13*_*i*_ is coded 0 for non-parents/parents of older children and 1 for parents who have at least one child in the 0–12 age bracket. We focus on this age range as parents of primary school age children are more likely to be impacted by a loss of childcare and by home schooling. β*_3_* captures the interaction between entering lockdown and parental status.

All outcomes are measured using ordinal scales but are treated as cardinal in line with the generally accepted approach to measuring subjective wellbeing in the empirical literature which assumes that Likert scales may be treated as continuous once individual fixed effects are accounted for.^10^ By way of robustness check, we re-estimate Eq. 1 using an ordered logit fixed effects model in Supplementary Table 5 and find no material differences in the results. Robust standard errors, clustered at the individual level, are employed throughout in accordance with Moulton (1990).

The Benjamini–Hochberg (1995) method is used to control for the false discovery rate (the proportion of significant results that represent false positives). *P*-values controlling for multiple testing are generated as follows: (1) The *p*-values from the 60 tests conducted for the analysis (see Table 3; 15 main effect analyses and 45 interaction analyses) are ranked from smallest to largest; (2) each *p*-value is compared to a critical value ([*i/m*]*Q), where *i* is the rank, *m* the total number of tests, and *Q* is the false discovery rate of 0.10; (3) *p*-values are deemed significant if they are smaller than the *p*-value Benjamini–Hochberg critical value at the relevant threshold (i.e., *p* < 0.05, *p* < 0.01, and *p* < 0.001).

## Results

### Descriptives

Bivariate correlations between the dependent variables are presented in Supplementary Table 6. The means of the raw outcome scores are presented in Table 2. The descriptives suggest that, on balance, entering lockdown does not appear to adversely affect worker wellbeing. The fixed effects models which are summarised in Table 3 formally tests this hypothesis.

**TABLE 2:** Mean outcome scores (standard deviation in parentheses).

Outcome	Wave 1 (*n*: 574–621)	Wave 2 (*n*: 585–620)
**Cognitive wellbeing**		
Life satisfaction (0–10)	6.62 (1.81)	6.56 (1.87)
Homelife satisfaction (0–10)	7.17 (2.04)	6.95 (2.05)
Job satisfaction (0–10)	5.97 (2.15)	6.11 (2.21)
**Emotional wellbeing**		
Global positive affect (0–6)	2.54 (1.08)	2.51 (1.13)
Global negative affect (0–6)	1.55 (1.15)	1.49 (1.16)
Experiential positive affect (0–6)	2.90 (0.92)	2.97 (0.99)
Experiential negative affect (0–6)	2.11 (0.761)	2.00 (0.697)
Affective commitment (1–5)	2.98 (1.01)	3.17 (1.02)
**Psychological wellbeing**		
Work stress (1–5)	3.08 (1.02)	3.06 (1.01)
Disengagement (1–4)	2.47 (0.57)	2.39 (0.58)
Exhaustion (1–4)	2.52 (0.56)	2.41 (0.54)
Relatedness (1–7)	4.95 (1.08)	5.06 (1.04)
Competence (1–7)	4.97 (1.03)	5.01 (0.99)
Autonomy (1–7)	4.43 (1.10)	4.53 (1.02)
Mental health (1–5)	3.61 (0.89)	3.64 (0.87)

**TABLE 3:** Fixed effects regression–standardised coefficients.

Variable	Whole sample (*n*: 1,159–1,241)	Wave*Homeworker (*n*: 1,152–1,233)	Wave*Gender (*n*: 1,149–1,231)	Wave*ParentU13 (*n*: 1,159–1,241)
**Cognitive wellbeing**				
Life satisfaction	−0.035 (0.036)	0.020 (0.094)	−0.051 (0.072)	−0.002 (0.088)
Homelife satisfaction	−0.108** (035)	−0.017 (0.094)	−0.010 (0.072)	0.040 (0.080)
Job satisfaction	0.059 (0.036)	0.092 (0.101)	−0.133 (0.073)	0.095 (0.093)
**Emotional wellbeing**				
Global positive affect	−0.025 (0.035)	−0.009 (0.091)	−0.130 (0.071)	−0.015 (0.088)
Global negative affect	−0.051 (0.032)	0.019 (0.077)	0.138 (0.063)	−0.014 (0.075)
Experiential positive affect	0.066 (0.042)	−0.002 (0.104)	0.032 (0.086)	0.038 (0.105)
Experiential negative affect	−0.150** (0.053)	−0.336** (0.121)	−0.023 (0.108)	0.097 (0.128)
**Psychological wellbeing**				
Work stress	−0.014 (0.032)	0.040 (0.083)	0.048 (0.066)	−0.070 (0.077)
Disengagement	−0.135*** (0.032)	0.006 (0.084)	0.180** (0.064)	0.047 (0.080)
Exhaustion	−0.198*** (0.030)	0.039 (0.073)	0.077 (0.061)	0.030 (0.069)
Relatedness	0.099*** (0.028)	−0.031 (0.072)	0.059 (0.057)	−0.057 (0.065)
Competence	0.043 (0.032)	−0.033 (0.076)	−0.094 (0.065)	0.037 (0.078)
Autonomy	0.093** (0.030)	0.055 (0.076)	−0.118 (0.058)	−0.016 (0.072)
Affective commitment	0.190*** (0.031)	0.052 (0.073)	−0.073 (0.061)	0.110 (0.074)
Mental health	0.029 (0.034)	−0.148 (0.084)	0.152 (0.072)	−0.017 (0.017)

*** p < 0.01, ** p < 0.05, * p < 0.1. Adjusted p-values are significant at the threshold identified (p < 0.05, p < 0.01, p < 0.001) after controlling for multiple testing (Benjamini–Hochberg procedure); Standardised variables used throughout. Robust clustered standard errors in parentheses; Wave coded 0 for Wave 1 and 1 for Wave 2. Homeworker coded 0 for non-homeworkers and 1 for homeworkers. Binary gender variable is employed which codes females 0 and males 1 and omits “non-binary/other” responses (6 respondents). Parent is coded 0 for non-parents or parents who do not have a child in the 0–12 age bracket brackets and 1 for parents with at least one child in the 0–12 age bracket.

### Fixed Effects Model of Within-Worker Changes

A linear fixed-effect model is estimated to examine changes in within-worker wellbeing associated with the COVID-19 restrictions. The main effect for each outcome is presented in Column 2 of Table 3. Effect sizes range from just under 0.1 standard deviations to just over 0.3 standard deviations. The results show that, on average, the impact of the COVID-19 restrictions on worker wellbeing is moderately positive. Adjusting for multiple hypothesis testing, we find that 9 of the 15 outcomes reach conventional levels of significance, with the restrictions having a negative impact on just one outcome (home life satisfaction) and a positive impact on 6 outcomes (experiential negative affect, disengagement, exhaustion, relatedness, autonomy, and affective commitment).

Although lockdown is associated with a moderate reduction in home life satisfaction, life satisfaction and global affect are relatively unaffected by the restrictions. The significant reduction in the frequency of negative emotions experienced at work the previous day suggests that experiential measures may be more sensitive to changes in contextual cues. Analysing each emotion individually (see Supplementary Tables 7, 8) indicates that the reduction is driven by a moderate decrease in high activation negative emotions, in particular anxiety (−0.120 sd; *p* = 0.026), tension (−0.149 sd; *p* = 0.008), and nervousness (−0.103 sd; *p* = 0.058).

Table 3 also shows that, somewhat surprisingly, the COVID-19 restrictions do not affect stress levels. Analysing the individual sources of stress cited by workers (see Supplementary Tables 9, 10), shows that the number of workers who are stressed by their *commute* (−0.38 sd; *p* < 0.001) or work-related *travel* (−0.17 sd; *p* = 0.008) falls sharply relative to the pre-COVID-19 period. *Personnel* issues are also less of a problem, with fewer workers citing their *bosses* (Beta = -0.10 sd; *p* = 0.045), *clients* (−0.15 sd; *p* < 0.001), or *co-workers* (−0.23 sd; *p* < 0.001) as a source of stress compared to before lockdown. Interestingly, fewer workers are stressed out by meeting *deadlines* (−0.26 sd; *p* < 0.001) or long *working hours* (−0.12 sd; *p* = 0.008) during lockdown, a result which aligns with the increased tendency to feel “relaxed” and “laidback” at work during lockdown as reported in Supplementary Tables 7, 8. However, the proportion of workers stressed about job security rises by 0.20 sd (*p* < 0.001).

Contrary to our priors, there is no evidence that lockdown is associated with increased psychological distress. Self-rated mental health remains stable, while the risk of burnout (captured by the disengagement and exhaustion outcomes) diminishes significantly. Supplementary Table 11 shows the standardised coefficients for each of the disengagement and exhaustion sub-scale items, of which 11 out of 16 improve significantly during lockdown. The largest improvements are found for exhaustion, with workers reporting significant reductions in the extent to which they feel tired before arriving at work (−0.21 sd; *p* < 0.001), need more time to relax after work (−0.22; *p* < 0.001) and feel emotionally drained during work (−0.12 sd; *p* < 0.001). They also report an improvement in the extent to which they feel energised at work (−0.14 sd: *p* < 0.001) and have sufficient energy for leisure activities (+0.22 sd; *p* < 0.001) relative to pre-COVID-19. The decrease in disengagement during lockdown reported in Table 3, is largely driven by a reduction in the extent to which workers speak negatively about their work (−0.22 sd; *p* < 0.001) or feel disconnected from it (−0.14 sd; *p* < 0.001) and increased levels of engagement in the work itself (+0.13 sd; *p* < 0.001) as reported in Supplementary Table 11.

Table 3 also shows that workers report improvements in the extent to which their basic psychological needs of relatedness and autonomy are met at work during lockdown, although the effect sizes are generally small. An analysis of sub-scale items (see Supplementary Table 12) reveals that the improvement in relatedness is driven by an increased sense of co-workers as friends (+0.09 sd; *p* < 0.001), who care about the worker (+0.16 sd; *p* < 0.001) and who take his/her feelings into consideration (+0.28 sd; *p* < 0.001). The improved autonomy score reflects greater freedom to express opinions (+0.11 sd; *p* < 0.001) and make inputs (+0.08 sd; *p* < 0.001) at work. Table 3 also shows that entering lockdown is associated with a moderate strengthening of the emotional bond between workers and their organisations, as measured by affective commitment.

Next, we estimate Eq. 2 to ascertain whether the COVID-19 restrictions differentially impact workers who worked from home during lockdown or continued to work from their usual workplace. The standardised coefficients for the wave*WFH interaction are set out in Column 3 of Table 3 (base = non-homeworker) and the marginal effects are depicted in Supplementary Table 13. We find just one main effect. Homeworkers report a greater decline in negative emotions experienced the previous day during lockdown than non-homeworkers. As Supplementary Table 9 shows, this is driven by homeworkers’ experiencing larger reductions in 5 (of 8) negative emotions than non-homeworkers, with the largest effects found for “despondent” (−0.44 sd; *p* < 0.001), “nervous” (−0.44 sd; *p* < 0.001), and “dejected” (−0.33 sd; *p* < 0.001).

We next investigate heterogeneity by gender by estimating Eq. 3. Column 4 of Table 3 contains the standardised coefficients for the wave*gender interaction (base = female). The marginal effects are set out in Supplementary Table 14. We find one main effect. Contrary to expectations, women do not appear to cope worse with the COVID-19 restrictions than men and women experience a larger reduction in disengagement during lockdown than men.

Finally, we estimate Eq. 4 to test the hypothesis that parents of young children (<13 years old)^11^ are more likely to experience adverse wellbeing consequences during lockdown due to the imposition of additional childcare or home-schooling burdens. Column 5 of Table 3 depicts the standardised coefficients for the wave*parentU13 interaction (base = non-parent of U13 child). For marginal effects see Supplementary Table 15. Contrary to our priors, we find no evidence that lockdown is experienced significantly differently by parents of young children.^12^ In sum, the heterogeneity analyses reveal few significant differences by homeworking, gender or parental status.

## Discussion

In contrast to other COVID-19 wellbeing studies, this study demonstrates that lockdown is not necessarily a negative experience for full-time workers with a high level of job security, income protection, and low physical exposure to the virus.^13^ Life satisfaction and overall emotional wellbeing are relatively unaffected by the first wave of COVID-19 restrictions. This may reflect relatively low baseline scores in this sample, which may dilute the impact of the COVID-19 shock.^14^ It may also reflect a data collection window which is too narrow to register lockdown-induced wellbeing changes using global measures which are more suited to capturing the effects of more enduring life events, such as parental death or unemployment.

Sample composition may also play a role. Employed individuals are likely, on average, to be healthier, both in terms of physical and mental health, than individuals who are out of the labour force (Egan et al., 2016). This may reduce the susceptibility of employed individuals to COVID-19 and lockdown related stress.^15^ However, while our sample may, on average, be healthier than the general population, there is evidence of considerable intra-sample physical and mental health heterogeneity, which mitigates against the possibility of overly positive findings.^16^ We excluded self-employed and part-time workers, as well as those no longer working due to COVID-19, are from our sample, thus eliminating groups of workers who may have been economically impacted by the pandemic. Furthermore, 87% of the respondents live with someone, a factor which has been found to increase the likelihood of “enjoying” lockdown (Fancourt et al., 2020).

An alternative explanation for the lack of significant changes in life satisfaction or global affect between the two periods is psychological adaptation. Research on “adaptive preferences” shows that individuals scale down their expectations to avoid disappointment when faced with adverse conditions (White, 2009). The timing of the wave two survey (6–11 weeks into lockdown) may have given workers sufficient time to adapt to the initial shock of lockdown. The relative stability in satisfaction and global affect may therefore mask a previous dip and subsequent reversion to pre-pandemic “set point” levels (Lykken and Tellegen, 1996).

The results provide some limited evidence that COVID-19 restrictions may not affect all workers equally. Contrary to other studies (e.g., Lyttelton et al., 2020), we find no evidence that parents of young children or women fare worse during lockdown. In fact, women report a larger decrease in disengagement than men. Similar to Zacher and Rudolph (2021), we find that homeworkers cope better emotionally with lockdown compared to non-homeworkers. Despite the sudden, largely involuntary shift to homeworking and the extraordinary pandemic-related backdrop of school closures, homeworkers report a larger decrease in the frequency with which they experience negative emotions at work. Zacher and Rudolph (2021) caution that the reduction in high-activation negative emotions that they identify could be off-set by an increase in (unmeasured) low-activation negative emotions. However, utilising measures of both high and low activation emotions, we find no evidence that entering lockdown is associated with a significant increase in low-activation emotions for homeworkers, raising the possibility that the decrease in negative affect revealed by both studies is not a measurement artefact but may instead reveal something more fundamental about the lived experience of homeworking during the pandemic. Finally, we find that entering lockdown is associated with a moderate drop in homelife satisfaction. Unlike Möhring et al. (2021), however, this finding holds for the entire sample, not just homeworkers.

In relation to psychological distress, somewhat surprisingly, we find no evidence of a deterioration in self-rated mental health during lockdown. This may reflect sample composition. The sample contains a low share of young adults, ethnic minorities and less educated workers, all of whom have been shown to be particularly vulnerable to COVID-19-induced mental health issues (e.g., Fancourt et al., 2020). The results also reveal a significant reduction in burnout symptoms, which is largely driven by reduced levels of exhaustion. Workers report feeling less tired before arriving at work and having more energy for leisure activities after work, findings which likely reflect reduced commuting time, but which may also signal pre-existing high levels of adaptive coping skills in our sample or a perceived reduction in job demands and/or increase in leisure opportunities on the part of respondents during the period of COVID-19 restrictions (Shoman et al., 2021). Workers are more engaged in their work and have a more positive attitude towards it. There is, however, limited evidence of heterogeneity, with men reporting significantly lower reductions in disengagement during lockdown than women.

To the best of our knowledge this is the first study to examine the impact of the COVID-19 restrictions on work-related psychological wellbeing. On the whole, we find positive effects. Workers feel more able to express their opinions during lockdown. They feel they have a greater input into their jobs and report feeling a greater sense of accomplishment from working and learning new skills during lockdown. Somewhat counterintuitively, workers feel closer to their colleagues and feel more cared for and listened to during lockdown, a finding which may reflect the “we’re all in this together” message propagated by the United Kingdom government at the start of COVID-19. Workers also report a stronger sense of emotional attachment to their organisations relative to the pre-COVID-19 period.

The study has some limitations which could be addressed by future research. The first area of potential concern relates to the selective nature of our sample. While the evidence that “professional” survey participants differ demographically and attitudinally from other survey participants is mixed (Hillygus et al., 2014; Huff and Tingley, 2015), our participants may differ systematically from the “average” worker (e.g., higher proportion of women and graduates), which detracts from wider generalisability. An obvious direction for future research is to target a more ethnically and socioeconomically diverse online sample and/or to extend our survey to a field setting. Secondly, the use of a fixed effects model, while econometrically appropriate, eliminates potentially policy-relevant sources of heterogeneity. Future research could tease out the relationship between additional covariates and homeworking preferences and/or effectiveness. Finally, the outcome variables are subjective, self-rated scales, which may raise concerns about self-report and recall bias. While including additional time points would partially address this, combining objective measures with self-rated data, would strengthen validity. The study would also benefit from the inclusion of additional waves of data to examine the longer-term impact of the pandemic and involuntary homeworking on wellbeing.

Decisions around appropriate pandemic responses require high-quality information on the potential psychological and emotional cost for society (Layard et al., 2020). Thus, this study has important implications for governments and employers. By utilising multiple measures to capture the lived reality of one such policy response (lockdown) for full-time workers and by demonstrating the heterogeneity in experiences, this study makes a valuable contribution to this debate. For example, the significant reduction in negative emotions suggests that experiential affective measures may play a role in assessing the wellbeing effects of pandemic response policies.

One by-product of the COVID-19 restrictions, which is likely to outlive the pandemic, is the global shift to homeworking. This study is one of few that captures the lived experience of homeworking and in particular, the lived experience of workers who have no prior experience of homeworking and who may not otherwise have chosen to do so. The results suggest that homeworkers may, on balance, feel less unhappy at work. Whether this wellbeing improvement is a novelty effect which will erode over time as workers adapt to the “new normal” or whether it is a feature of homeworking under “normal” circumstances, is an important policy question which is currently unknown and which warrants further investigation. Our study represents an important first step in this direction.

## Data Availability Statement

The raw data supporting the conclusions of this article will be made available by the authors, without undue reservation.

## Ethics Statement

The studies involving human participants were reviewed and approved by UCD Human Research Ethics Committee. The patients/participants provided their written informed consent to participate in this study.

## Author Contributions

DP: conceptualisation, investigation, formal analysis, and writing–original draft preparation. MD and OD: conceptualisation, methodology, and writing–review and editing. LD: conceptualisation, methodology, writing–review and editing, and funding acquisition. All authors contributed to the article and approved the submitted version.

## Conflict of Interest

The authors declare that the research was conducted in the absence of any commercial or financial relationships that could be construed as a potential conflict of interest.

## Publisher’s Note

All claims expressed in this article are solely those of the authors and do not necessarily represent those of their affiliated organizations, or those of the publisher, the editors and the reviewers. Any product that may be evaluated in this article, or claim that may be made by its manufacturer, is not guaranteed or endorsed by the publisher.

## Footnotes

1 Personality profile may also play a mitigating role. For example, Pishghadam and Sahebjam (2012) find that high neuroticism and low extraversion scores predict a higher incidence of emotional exhaustion and depersonalisation, both key aspects of burnout, amongst teachers.

2 The Prolific United Kingdom database includes participants who are mainly white, full-time workers. In total, 55% of the panel are female. A total of 75% are in the 20–40 age bracket and 50% hold university degrees. Participants were paid an hourly rate of £8.20.

3 During wave two, three workers are based in Ireland. Excluding these workers does not materially affect the results.

4 While we cannot rule out the possibility that non-COVID-19 related seasonality may have influenced our results, previous research has found minimal effects of seasonality in relation to measures of emotional distress during the pandemic (e.g., Daly et al., 2020).

5 Six workers were on maternity leave. The remaining 114 were furloughed or unemployed. Unemployed workers are excluded due to an extensive literature linking unemployment with systematically lower wellbeing, e.g., Winkelmann and Winkelmann (1998) and Lucas et al. (2004).

6 Disengagement and Exhaustion contain 83 and 72 incomplete observations, respectively, which are excluded from the analysis.

7 While single-item measures correlate highly with longer life-satisfaction scales (Cheung and Lucas, 2014), as a robustness check we also use a multi-faceted domain measures of job satisfaction–the Abridged Job Descriptive Index (AJDI) (Stanton et al., 2002).

8 We employ the scoring system used in Demerouti et al. (2010) in this study (see Supplementary Table 3).

9 Just 3% of workers report working from home on a part-time basis during lockdown.

10 Ferrer-i-Carbonell and Frijters (2004) show that results are not sensitive to the choice of OLS method–a finding replicated in several studies. Recent COVID-19 studies treat wellbeing cardinally (e.g., Zacher and Rudolph, 2021). Baetschmann et al. (2015 p. 685) point out that “there is no consensus in the past literature on how to implement a fixed effects estimator for the ordered logit model.”

11 23.4% of the sample or 145 workers have children under the age of 13.

12 To investigate whether this finding is related to sample size effects, we re-estimate the regression using (i) all parents (49.7% of the sample) and (ii) whether or not workers live in a house containing children (52.2% of the sample). The results do no change materially.

13 Just 31% of the sample are financially negatively impacted by COVID-19 and only 6% have contracted COVID-19 or had to quarantine. The sample contains a high number of highly educated, full-time workers, 96% of whom are on permanent contracts.

14 Average life satisfaction in the United Kingdom is 7.7 versus a baseline level of 6.6 in this sample. Layard et al. (2020) also report a substantial decrease in life satisfaction in their United Kingdom sample prior to lockdown. Hudson et al. (2019) report average global positive and negative affect of approximately 4 and 2.5, respectively, versus our baseline levels of 2.5 and 1.5.

15 For example, Robinson et al. (2022) found that those with pre-existing physical (but not mental) health conditions were at greater risk of adverse mental health effects associated with the pandemic.

16 Just under one quarter of the sample reported having a chronic physical or mental health condition at wave one.
