# Supplementary material for: Worker Stress, Burnout, and Wellbeing Before and During the COVID-19 Restrictions in the United Kingdom
Source: Front Psychol. 2022 Apr 5;13:823080. doi: 10.3389/fpsyg.2022.823080 (PMC9040160; doi:10.3389/fpsyg.2022.823080)
Supplement: Supplementary file 1 [file Data_Sheet_1.docx]

**Figure S1. Distribution of Responses by Month of Collection (Nov 2019 – July 2020)**

**Figure S2: Comparison of Sample Characteristics with Wheatley (2020)^[[1]](#footnote-2)^**

|  | WHEATLEY (2020) | **OUR STUDY** |
| --- | --- | --- |
| *Gender* |  |  |
| Women | 55% | **64%** |
| Men | 45% | **36%** |
| *Age* |  |  |
| 16-24 | 13% | **4%** |
| 25-29 | 11% | **17%** |
| 30-39 | 24% | **38%** |
| 40-49 | 26% | **25%** |
| 50-59 | 19% | **13%** |
| 60-69 | 6% | **2%** |
| 70 or over | 1% | **0%** |
| *Education* |  |  |
| No qualifications | 6% | **0%** |
| GCSE or above | 26% | **17%** |
| A Levels or equivalent | 37% | **21%** |
| Degree or above | 33% | **62%** |
| *Country of Residence* |  |  |
| England | 82% | **83%** |
| Wales | 6% | **3%** |
| Scotland | 8% | **9%** |
| NI | 4% | **5%** |
| *Long term condition / disability^[[2]](#footnote-3)^* | 23% | **23%** |
| *Relationship status* |  |  |
| Single / Divorced / Widowed | 49% | **26%** |
| In a relationship | 51%^[[3]](#footnote-4)^ | **74%** |
| *Annual Household income* | £26,600 | **£24,000 - £36,000** |
| Permanent Contract | 93% | **96%** |
| Overall Life Satisfaction | 5.2 | **6.5** |
| Job Satisfaction | 5.3 | **6.0** |

**Table S3: Full Description of Outcome Variables**

| **VARIABLE** | **QUESTION IN ABBREVIATED FORM** | **ANSWER SCALE** |
| --- | --- | --- |
| Overall Life Satisfaction | Respondents are asked to rate their overall life satisfaction | 0= “Completely Dissatisfied”; 10 = “Completely Satisfied” |
| Home Life Satisfaction | Respondents are asked to rate their overall life satisfaction | 0= “Completely Dissatisfied”; 10 = “Completely Satisfied” |
| Overall Job Satisfaction | Respondents are asked to rate their overall life satisfaction | 0= “Completely Dissatisfied”; 10 = “Completely Satisfied” |
| Self-Rated Mental Health | Respondents are asked to rate their overall mental health status | 1= “Very Bad”  5= “Very Good” |
| Work-related Stress | Respondents are asked to rate the stress levels associated with their job | 1 = “Not at all stressful”  5 = “Extremely stressful” |
| Sources of work stress | Respondents are asked to indicate what aspects of work are causing them the most stress | 15 response options e.g. “clients” and “other” |
| Multi-dimensional Job Satisfaction | **Job Descriptive Index (JDI)(Stanton et al., 2002)**  Respondents are asked the extent to which 6-8 adjectives relating to each of the following dimensions of job satisfaction: satisfaction with the job in general; pay; supervision; promotion opportunities; people encountered at work; the work itself; accurately describes it **e**.g. “*Annoying”* | 3 options for each question:  ‘Yes’ if the word is an accurate descriptor; ‘No’ if the word is not an accurate descriptor and ? if the respondent is unsure |
| Global Affect (Feelings experienced at work) | **IWP Multi-Affect Indicator (Parker & Warr, 2010; 2016)**  Respondents are asked to indicate the extent to which they experienced 16 different emotions at work during the previous month | 7-point Likert scale:  1= “Never (0% of the time”;  4= “About half of the time” (41% to 60%);  7 = “Always” (100% of the time”)  (Recoded to 0-6 scale in our study for ease of comparison with DRM) |
| Experiential Affect (Feelings experienced over 3 episodes the previous day) | **Day Reconstruction Method (DRM) – Kahneman et al., 2004)**  Respondents are asked to indicate the extent to which they experienced 16 emotions during three episodes the previous day | 0 = “Not at all”  6 = “Very much” |
| Engagement / Exhaustion | **16-item Oldenburg-Burnout Inventory (OLBI) - Demerouti & Bakker (2008); Demerouti, Mostert & Bakker (2010)^[[4]](#footnote-5)^**  Respondents are asked to agree / disagree with 16 statements e.g.  *“There are days when I feel tired before I arrive at work”* | 1 = “Strongly Agree”  4 = “Strongly Disagree” |
| Relatedness / Competence / Autonomy | **21-itemBasic Psychological Needs Satisfaction at Work Scale (Deci et al., 2001); Ilardi et al,1993; Kasser, Davey & Ryan, 1992)**  Respondents are asked to indicate the extent to which 21 statements applied to them at work over the previous month. *E.g. “When I am at work..I feel pressured at work”.* | 1 = “Not at all true”  7 = “Very much true” |
| Organisational Commitment (Affective Commitment) | **6-item Affective Commitment Scale (Meyer & Allen,1997)**  Respondents are asked to agree / disagree with six statements *e.g. “I do not feel like ‘part of the family’ at my organization”* | 1 = “Strongly Disagree”  5= “StronglyAgree” |

**Table S4. Standardised Main Effects (SD) by Outcome – comparison of OLS, Fixed Effects (FE) and Random Effects (RE) Models**

| **Variable** | **Coefficient (SD change)OLS** | **Coefficient (SD change)FE** | **Coefficient (SD change)RE** |
| --- | --- | --- | --- |
| Life Satisfaction | -.036  (.036) | -.036  (.036) | -.036  (.036) |
| Homelife Satisfaction | -.109***  (.035) | -.108***  (.036) | -.109***  (.036) |
| Job Satisfaction | .063*  (.037) | .059  (.037) | .061*  (.037) |
| Global Positive Affect | -.032  (.035) | -.025  (.035) | -.027  (.035) |
| Global Negative Affect | -.062*  (.032) | -.052  (.032) | -.055*  (.032) |
| Experiential positive affect | .073  (.042) | .066  (.043) | .070  (.043) |
| Experiential negative affect | -.164***  (.053) | -.150***  (.053) | -.162***  (.053) |
| Work Stress | -.017  (.032) | -.014  (.032) | -.015  (.032) |
| Disengagement | -.145***  (.033) | -.135***  (.032) | -.138***  (.032) |
| Exhaustion | -.211***  (.031) | -.197***  (.030) | -.201***  (.030) |
| Relatedness | .096***  (.028) | .099***  (.028) | .098***  (.028) |
| Competence | .045  (.032) | .043  (.032) | .044  (.032) |
| Autonomy | .105***  (.030) | .092***  (.030) | .096***  (.030) |
| Affective Commitment | .179***  (.031) | .190***  (.031) | .187***  (.031) |
| Mental Health | .035  (.035) | .029  (.034) | .032  (.034) |

**** p<0.01, ** p<0.05, * p<0.1Non-adjusted p-values. Standardised variables used throughout. Robust clustered standard errors in parentheses. Not adjusted for multiple inference.*

**Table S5: Ordered Logit FE model – Standardised Main Effect (SD) by Outcome**

| **Variable** | **Coefficient (SD change)** | **Log Conditional Likelihood** |
| --- | --- | --- |
| Life Satisfaction | -.119  (.120) | -474.967 |
| Homelife Satisfaction | -.341**^***^**  (.113) | -550.515 |
| Job Satisfaction | .181  (.112) | -610.511 |
| Global Positive Affect (IWP) | -.083  (.108) | -2413.253 |
| Global Negative Affect (IWP) | -.185  (.114) | -2103.043 |
| Experiential Positive Affect (DRM) | .161  (.107) | -9490.099 |
| Experiential Negative Affect (DRM) | -.296**^***^**  (.109) | -8515.229 |
| Work Stress | -.055  (.124) | -223.761 |
| Disengagement | -.488**^***^**  (.116) | -1021.604 |
| Exhaustion | -.750**^***^**  (.117) | -850.847 |
| Relatedness | .397**^***^**  (.114) | -1718.186 |
| Competence | .143**^***^**  (.107) | -1505.474 |
| Autonomy | .340**^***^**  (.108) | -1648.178 |
| Affective Commitment | .693**^***^**  (.117) | -1336.679 |
| Mental Health | .108  (.129) | -203.349 |

**** p<0.01, ** p<0.05, * p<0.1. Unadjusted p-values. Standardised variables used throughout. Robust clustered standard errors in parentheses. Estimated using Stata feologit command. Baetschmann, G., K. E. Staub, and R. Winkelmann 2015. Consistent estimation of the fixed effects ordered logit model. Journal of the Royal Statistical Society, Series A 178: 685-703.* [*https://doi.org/10.1111/rssa.12090*](https://doi.org/10.1111/rssa.12090)*.*

**Table S6: Pairwise Correlations between the outcome variables**

| Variables | **LS** | **JS** | **DRM PA** | **DRM NA** | **IWP PA** | **IWP NA** | **Stress** | **Disengage** | **Exhaust** | **Rel** | **Comp** | **Auton** | **AC** | **HLS** |
| --- | --- | --- | --- | --- | --- | --- | --- | --- | --- | --- | --- | --- | --- | --- |
| **(1) Life Satisfaction** | 1.000 |  |  |  |  |  |  |  |  |  |  |  |  |  |
| **(2) Job Satisfaction** | 0.566* | 1.000 |  |  |  |  |  |  |  |  |  |  |  |  |
| **(3) DRM Positive Affect** | 0.383* | 0.425* | 1.000 |  |  |  |  |  |  |  |  |  |  |  |
| **(4) DRM Negative Affect** | -0.125* | -0.198* | -0.218* | 1.000 |  |  |  |  |  |  |  |  |  |  |
| **(5) IWP Positive Affect** | 0.440* | 0.587* | 0.561* | -0.240* | 1.000 |  |  |  |  |  |  |  |  |  |
| **(6) IWP Negative Affect** | -0.408* | -0.526* | -0.417* | 0.308* | -0.523* | 1.000 |  |  |  |  |  |  |  |  |
| **(7) Stress** | -0.181* | -0.247* | -0.298* | 0.184* | -0.371* | 0.403* | 1.000 |  |  |  |  |  |  |  |
| **(8) Disengagement** | -0.354* | -0.672* | -0.413* | 0.195* | -0.581* | 0.493* | 0.146* | 1.000 |  |  |  |  |  |  |
| **(9) Exhaustion** | -0.385* | -0.571* | -0.472* | 0.261* | -0.626* | 0.634* | 0.498* | 0.633* | 1.000 |  |  |  |  |  |
| **(10) Relatedness** | 0.283* | 0.400* | 0.335* | -0.095* | 0.368* | -0.358* | -0.115* | -0.438* | -0.324* | 1.000 |  |  |  |  |
| **(11) Competence** | 0.346* | 0.537* | 0.381* | -0.135* | 0.497* | -0.503* | -0.129* | -0.625* | -0.487* | 0.528* | 1.000 |  |  |  |
| **(12) Autonomy** | 0.364* | 0.549* | 0.408* | -0.206* | 0.520* | -0.543* | -0.342* | -0.586* | -0.587* | 0.509* | 0.632* | 1.000 |  |  |
| **(13) Affective Commitment** | 0.287* | 0.565* | 0.336* | -0.096* | 0.412* | -0.342* | -0.047 | -0.652* | -0.401* | 0.481* | 0.535* | 0.541* | 1.000 |  |
| **(14) Homelife Satisfaction** | 0.730* | 0.358* | 0.320* | -0.086* | 0.322* | -0.296* | -0.138* | -0.209* | -0.267* | 0.200* | 0.228* | 0.244* | 0.162* | 1.000 |

**Table S7. Global Affect: Individual Emotions – Standardised Coefficients**

| **Variable** | **Whole sample** | **Wave*WFH** | **Wave*Gender** | **Wave*ParentU13** |
| --- | --- | --- | --- | --- |
| *High Activation Positive* |  |  |  |  |
| Excited | -.086^**^ (.038) | -.032 (.098) | -.040 (.076) | -.103 (.094) |
| Inspired | -.062 (.038) | -.054 (.098) | -.159^*^ (.075) | -.154^*^ (.087) |
| Enthusiastic | -.180^***^ (.039) | -.102 (.096) | .007 (.081) | -.081 (.092) |
| Joyful | -.065^*^ (.038) | -.153(.098) | -.050(.075) | .008(.090) |
| *Low Activation Positive* |  |  |  |  |
| Calm | .012 (.039) | -.038 (.095) | -.164^**^ (.079) | -.183^*^ (.095) |
| Relaxed | .094^**^ (.040) | .148 (.095) | -.130 (.083) | -.027 (.098) |
| At Ease | .054 (.041) | .081(.104) | -.142 (.086) | -.160 (.101) |
| Laid back | .072^*^(040) | .207^*^ (.104) | -.078 (.083) | .025 (.092) |
| *High Activation Negative* |  |  |  |  |
| Anxious | .061^*^ (.035) | -.024 (.083) | .080 (.074) | .127 (.122) |
| Worried | .078^*^ (.038) | -.058 (.094) | .138^*^ (.077) | .245^***^ (.091) |
| Tense | -.100^***^ (.038) | .030 (.099) | .107 (.075) | .210^**^ (.088) |
| Nervous | .050 (.037) | .076 (.086) | -.012 (.073) | .032 (.085) |
| *Low Activation Negative* |  |  |  |  |
| Depressed | - .040 (.036) | .019 (.083) | .086 (.074) | -.037 (.083) |
| Dejected | -.180^***^ (.039) | -.106 (.097) | .249^***^ (.077) | .038 (.082) |
| Despondent | -.083^**^ (.039) | .030 (.090) | .081 (.077) | .067 (.083) |
| Hopeless | -.090^**^ (.038) | .034 (.090) | .161^**^ (.077) | .089 (.085) |

**Table S8. Experiential Affect: Individual Emotions – Standardised Coefficients**

| **Variable** | **Whole sample** | **Wave*WFH** | **Wave*Gender** | **Wave*ParentU13** |
| --- | --- | --- | --- | --- |
| *High Activation Positive* | -.023 (.049) | -.010 (.115) | .029 (.099) | -.127 (.112) |
| Excited | .036 (.047) | .122 (.114) | .019 (.098) | -.124 (.113) |
| Inspired | -.011 (.046) | .017 (.111) | .011 (.092) | .112 (.111) |
| Enthusiastic | .037 (.047) | -.154 (.116) | .010 (.096) | -.130 (.116) |
| Joyful |  |  |  |  |
| *Low Activation Positive* | .081^*^ (.044) | .125 (.102) | .014 (.095) | -.088 (.105) |
| Calm | .117^**^ (.045) | .180^*^ (.106) | -.047 (.094) | -.131 (.093) |
| Relaxed | .089^*^ (.045) | .046 (.110) | -.009 (.093) | -.007 (105) |
| At Ease | .109^**^ (044) | .041 (.113) | .173^*^ (092) | -.108 (102) |
| Laid back |  |  |  |  |
| *High Activation Negative* | -.120^**^ (.026) | -.265^**^ (.127) | -.170 (.109) | .246^**^ (.122) |
| Anxious | -.090 (.055) | -.318^**^ (.130) | -.049 (.112) | .205^*^ (.122) |
| Worried | -.149^**^ (.055) | -.263^*^ (.137) | -.127 (.114) | .192 (.131) |
| Tense | -.103^*^ (.054) | -.440^***^ (.126) | -.248^**^ (.114) | .141 (.120) |
| Nervous |  |  |  |  |
| *Low Activation Negative* | .014 (.055) | -.150 (.138) | -.042 (.113) | -.083 (.124) |
| Depressed | -.100^*^ (.055) | -.333^***^ (.126) | .204^*^ (.114) | .038 (.126) |
| Dejected | -.052 (.056) | -.447^***^ (.131) | .195^*^ (.113) | .129 (.133) |
| Despondent | -.021 (.054) | -.168 (.139) | -.083 (.110) | .047 (.122) |
| Hopeless | -.023 (.049) | -.010 (.115) | .029 (.099) | -.127 (.112) |

**Table S9. Sources of Stress - Proportions**

| **Sources of Stress** | **Wave 1 Proportion** | **Wave 2 Proportion** | **t-score** |
| --- | --- | --- | --- |
| Clients | 31.2% | 24.4% | 3.41 |
| Co-Workers | 33.4% | 23.0% | 4.75 |
| Deadlines | 42.1% | 29.7% | 5.47 |
| Work Life Balance | 25.7% | 27.3% | -0.80 |
| Boss / Supervisor | 30.9% | 26.5% | 2.01 |
| Commute | 19.3% | 6.3% | 7.63 |
| Work-related travel | 6.6% | 2.8% | 3.38 |
| Revenue Generation | 9.1% | 9.6% | -0.35 |
| Long Hours | 22.7% | 17.7% | 2.64 |
| Job Security | 7.7% | 14.0% | -4.00 |

**Table S10. Sources of Stress – Regression Analysis (Standardised Coefficients)**

| **Dependent Variable: Source of Stress** | **Wave 1 (n = 1,242)** |
| --- | --- |
| Clients | -.150^***^ (.044) |
| Co-Workers | -.232^***^ (.048) |
| Deadlines | -.258^***^ (.047) |
| Work Life Balance | .036 (.045) |
| Boss / Supervisor | -.096^*^ (.047) |
| Commute | -.378^***^ (.049) |
| Work-related travel | -.174^***^ (.051) |
| Revenue Generation | .016 (.047) |
| Long Hours | -.124^***^ (.047) |
| Job Security | .201^***^ (.050) |

**Table S11.OLBI Burnout Scale: Individual Scale Items – Standardised Coefficients**

| **Variable** | **Whole sample** | **Wave*WFH** | **Wave*Gender** | **Wave*ParentU13** |
| --- | --- | --- | --- | --- |
| *Disengagement* |  |  |  |  |
| I always find new and interesting aspects in my job | .044 (.037) | -.065 (.092) | .038 (.075) | .164^*^ (.085) |
| It happens more and more often that I talk about my work in a negative way (R) | .221 (.034) | -.110 (.091) | -.111 (.073) | -.157^*^ (.085) |
| I can tolerate the pressure of my work very well | .026 (.037) | .091 (.092) | .051 (.075) | .089 (.087) |
| Lately, I tend to think less at work and do my job almost mechanically (R) | .087^**^ (.041) | .042 (.101) | -.205^**^ (.085) | -.117 (.091) |
| I find my work to be a positive challenge | -.091^**^ (.037) | .064 (.098) | .103 (.075) | .199^**^ (.088) |
| Over time, one can become disconnected from this type of work (R) | .137^***^ (.041) | .130 (.196) | -.117 (.088) | -.046 (.098) |
| Sometimes I feel sickened by my work tasks (R) | .125^***^ (.040) | -.128 (.106) | -.102 (.085) | -.169^*^ (.094) |
| This is the only type of work that I can imagine myself doing | .005 (.036) | -.080 (.093) | .128^*^ (.076) | .112 (.084) |
| I feel more and more engaged in my work | -.128^***^ (.040) | -.051 (.094) | .171^*^ (.084) | .160^*^ (.094) |
| *Exhaustion* |  |  |  |  |
| There are days when I feel tired before I arrive at work (R) | .215^***^ (.034) | .001 (.080) | .024 (.074) | -.119 (.008) |
| After work, I tend to need more time than in the past in order to relax and feel better (R) | .219^***^ (.040) | .081 (.096) | -.014 (.084) | -.247^***^ (.090) |
| During my work, I often feel emotionally drained (R) | .120^***^ (.040) | -.023 (.105) | -.052 (.081) | -.161^*^ (.093) |
| After working, I have enough energy for my leisure activities | -.220^***^ (.038) | -.022 (.096) | .044 (.081) | -.060 (.085) |
| After my work, I usually feel worn out and weary (R) | .160^***^ (.039) | -.062 (.085) | -.077 (.075) | .005 (.087) |
| Usually, I can manage the amount of my work well | -.051 (.045) | .042 (.109) | .171^*^ (.094) | -.030 (.097) |
| When I work, I usually feel energized | -.140^***^ (.038) | .125 (.089) | .084 (.081) | .106 (.093) |

**Table S12.Basic Psychological Needs Satisfaction at Work Scale: Individual Scale Items – Standardised Coefficients**

| **Variable** | **Whole sample** | **Wave*WFH** | **Wave*Gender** | **Wave*ParentU13** |
| --- | --- | --- | --- | --- |
| ***Relatedness*** |  |  |  |  |
| I really like my co-workers | .138 (.036) | .020 (.093) | -.059 (.074) | -.018 (.083) |
| I get along with people at work | .023 (.036) | .015 (.094) | .016 (.076) | -.023 (.081) |
| I pretty much keep to myself at work (R) | .039 (.036) | .122 (.081) | -.110 (.073) | .111 (.081) |
| I consider the people I work with to be my friends | .092^***^ (.032) | .031 (.084) | .128^*^ (.068) | -.008 (.070) |
| My feelings are taken into consideration at work | .279^***^ (.035) | .093 (.094) | -.186^**^ (.073) | -.036 (.080) |
| People at work care about me | .164^**^ (.034) | -.036 (.081) | -.099 (.068) | -.052 (.079) |
| There are not many people at work that I am close to (R) | -.117^***^ (.041) | .120 (.197) | -.211^**^ (.086) | -.071 (.096) |
| The people I work with do not seem to like me very much (R) | -.034 (.039) | .014 (.093) | .012 (.085) | .026 (.092) |
| People at work are pretty friendly towards me | .033 (.035) | .053 (.080) | -.083 (.072) | -.037 (.080) |
| ***Competence*** |  |  |  |  |
| I do not feel competent when I work | .063 (.042) | .184^*^ (.105) | -.103 (.087) | -.039 (.096) |
| People at work tell me I’m good at what I do | -.051 (.040) | .029 (.096) | -.048 (.082) | -.037 (.090) |
| I have been able to learn interesting new skills on my job | 066^*^ (.067) | .049 (.090) | -.027 (.075) | -.032 (.083) |
| Most days I feel a sense of accomplishment from working | .246^***^ (.034) | .057 (.084) | -.155^**^ (.069) | -.086 (.083) |
| On my job I don’t get much of a chance to show how capable I am (R) | .000 (.040) | .006 (.098) | .193^**^ (.083) | .014 (.094) |
| When I am working I often do not feel very capable (R) | .080^**^ (.037) | .034 (.090) | -.052 (.074) | .027 (.085) |
| ***Autonomy*** |  |  |  |  |
| I feel like I can make lots of inputs into how my job is done | .085^**^ (.037) | .038 (.095) | -.265^***^ (.076) | .037 (.088) |
| I feel pressured at work (R) | .000 (.038) | .036 (.090) | -.011 (.077) | -.093 (.084) |
| I am free to express my ideas and opinions on the job | .113^***^ (.038) | .176^*^ (.095) | -.100 (.078) | -.005 (.084) |
| When I’m at work I have to do what I am told (R) | .026 (.039) | .000 (.097) | -.078 (.081) | .020 (.100) |
| I feel like I can pretty much be myself at work | .041 (.037) | -.168^**^ (.085) | -.050 (.075) | -.116 (.083) |
| There is not much opportunity for me to decide how to go about my work (R) | .015 (.039) | -.115 (.095) | -.047 (.080) | .080 (.089) |

**Table S13. Marginal Effects by WFH (standardised)**

| **Variable** | **Non-Homeworkers** | **Homeworkers** | **Obs** |
| --- | --- | --- | --- |
| Life Satisfaction | -.054*(.085)* | -.034*(.039)* | 1,233 |
| Homelife Satisfaction | -.093*(.086)* | -.111**^***^** *(.039)* | 1,230 |
| Job Satisfaction | -.013 *(.094)* | .078**^**^** *(.050)* | 1,231 |
| Global Positive Affect (IWP) | -.015 (.082) | -.025 (.038) | 1,204 |
| Global Negative Affect (IWP) | -.068*(.068)* | -.049*(.037)* | 1,204 |
| Experiential Positive Affect (DRM) | .067*(.092)* | .064*(.048)* | 1,208 |
| Experiential Negative Affect (DRM) | .117*(.105)* | -.219*(.060)* | 1,208 |
| Work Stress | -.043*(.076)* | -.002*(.035)* | 1,224 |
| Disengagement | -.142**^*^** *(.070)* | -.135**^***^** *(.036)* | 1,152 |
| Exhaustion | -.229**^***^***(.065)* | -.189**^***^** *(.034)* | 1,163 |
| Relatedness | .123**^*^***(.065)* | .092**^***^** *(.032)* | 1,205 |
| Competence | .071*(.066)* | .038 *(.037)* | 1,205 |
| Autonomy | .054*(.049)* | .109**^***^** *(.023)* | 1,207 |
| Affective Commitment | .151**^**^** *(.064)* | .203**^***^***(.035)* | 1,214 |
| Mental Health | .148^*^ *(.075)* | .002*(.039)* | 1,227 |

**** p<0.01, ** p<0.05, * p<0.1. Robust Clustered Standard Errors. Standardised variables used throughout. P-values not adjusted for multiple inference.*

**Table S14. Marginal Effects by Gender (standardised)**

| **Variable** | **Women** | **Men** | **Obs** |
| --- | --- | --- | --- |
| Life Satisfaction | -.018 *(.048)* | -.069*(.053)* | 1,231 |
| Homelife Satisfaction | -.105^**^*(.046)* | -.115**^**^** *(.055)* | 1,228 |
| Job Satisfaction | .106^**^ *(.048)* | -.027*(.055)* | 1,228 |
| Global Positive Affect (IWP) | .023 *(.046)* | -.107^**^*(.053)* | 1,215 |
| Global Negative Affect (IWP) | -.103^**^*(.043)* | .034*(.045)* | 1,201 |
| Experiential Positive Affect (DRM) | .055*(.056)* | .088*(.065)* | 1,205 |
| Experiential Negative Affect (DRM) | -.139^*^**^*^** *(.069)* | -.163**^**^** *(.082)* | 1,205 |
| Work Stress | -.035 *(.041)* | .013*(.052)* | 1,222 |
| Disengagement | -.194**^***^***(.043)* | -.014*(.048)* | 1,149 |
| Exhaustion | -.225**^***^***(.040)* | -.148**^***^** *(.046)* | 1,160 |
| Relatedness | .078**^**^***(.038)* | .137**^***^***(.043)* | 1,202 |
| Competence | .076^*^ *(.043)* | -.017*(.048)* | 1,201 |
| Autonomy | .134**^***^** *(.040)* | .016*(.042)* | 1,204 |
| Affective Commitment | .211**^***^***(.040)* | .137**^***^** *(.046)* | 1,211 |
| Mental Health | -.028 *(.044)* | .124^**^*(.057)* | 1,224 |

**** p<0.01, ** p<0.05, * p<0.1. Robust Clustered Standard Errors. Standardised variables used throughout. Non-Adjusted p-values used throughout.*

**Table S15. Marginal effects for ParentU13**

| **Variable** | | **Non- Parents U13**  **Coefficient (SD)** | **ParentsU13**  **Coefficient (SD)** | **Obs** |
| --- | --- | --- | --- | --- |
| Life Satisfaction | | -.035*(.041)* | -.037*(.077)* | 1,241 |
| Homelife Satisfaction | | -.117^***^*(.041)* | -.077*(.068)* | 1,238 |
| Job Satisfaction | | .036*(.040)* | .132 *(.089)* | 1,238 |
| Global Positive Affect | | -.021*(.039)* | -.037*(.079)* | 1,225 |
| Global Negative Affect | | -.048*(.036)* | -.063*(.059)* | 1,211 |
| Experiential Positive Affect | | .057*(.048)* | .095*(.094)* | 1,215 |
| Experiential Negative Affect | | -.175^*^**^**^***(.059)* | -.076*(.113)* | 1,215 |
| Work Stress | | -.002 *(.036)* | -.068*(.064)* | 1,232 |
| Disengagement | | -.146**^***^***(.036)* | -.099*(.071)* | 1,159 |
| Exhaustion | | -.205**^***^***(.035)* | -.174**^***^***(.059)* | 1,170 |
| Relatedness | | .112**^***^***(.033)* | .055*(.056)* | 1,212 |
| Competence | | .038*(.036)* | .072 *(.069)* | 1,211 |
| Autonomy | | .096**^***^** *(.034)* | .080*(.065)* | 1,214 |
| Affective Commitment | | .165**^***^***(.036)* | .275**^***^***(.065)* | 1,221 |
| Mental Health | .033 *(.039)* | .015 *(.072)* | 1,234 |  |

**** p<0.01, ** p<0.05, * p<0.1.Non-adjusted p-values. Robust Clustered Standard Errors. Standardised variables used throughout.*

1. *Wheatley (2020) source: Understanding Society Waves 2, 4, 6 and 8. Data for Workers only. Excludes self-employed. ^2^ Assumed to refer to a chronic physical disability.*

   *3 Wheatley differentiates using marriage / civil partnership. This may underestimate workers in a relationship as workers who have never been married are designated as single although they may be in a (non-married) relationship.* [↑](#footnote-ref-2)
2. [↑](#footnote-ref-3)
3. [↑](#footnote-ref-4)
4. We employ the scoring system from Demerouti, Mostert & Bakker (2010) in this study. [↑](#footnote-ref-5)
